# Pharmacological targeting of the PI3K/mTOR pathway alters the release of angioregulatory mediators both from primary human acute myeloid leukemia cells and their neighboring stromal cells

**DOI:** 10.18632/oncotarget.971

**Published:** 2013-05-11

**Authors:** Håkon Reikvam, Ina Nepstad, Øystein Bruserud, Kimberley Joanne Hatfield

**Affiliations:** ^1^ Section for Hematology, Department of Clinical Science, University of Bergen, Norway; ^2^ Department of Medicine, Haukeland University Hospital, Bergen, Norway

**Keywords:** Acute myeloid leukemia, cytokines, mTOR, PI3K, Angiogenesis, Bone marrow microenvironment, Pharmacological intervention

## Abstract

Acute myeloid leukemia (AML) is a heterogeneous and aggressive malignancy with poor overall survival. Constitutive as well as cytokine-initiated activation of PI3K/Akt/mTOR signaling is a common feature of AML patients, and inhibition of this pathway is considered as a possible therapeutic strategy in AML. Human AML cells and different stromal cell populations were cultured under highly standardized in vitro conditions. We investigated the effects of mTOR inhibitors (rapamycin and temsirolimus) and PI3K inhibitors (GDC-0941 and 3-methyladenin (3-MA)) on cell proliferation and the constitutive release of angioregulatory mediators by AML and stromal cells.

Primary human AML cells were heterogeneous, though most patients showed high CXCL8 levels and detectable release of CXCL10, Ang-1, HGF and MMP-9. Hierarchical clustering analysis showed that disruption of PI3K/Akt/mTOR pathways decreased AML cell release of CXCL8-11 for a large subset of patients, whereas the effects on other mediators were divergent. Various stromal cells (endothelial cells, fibroblasts, cells with osteoblastic phenotype) also showed constitutive release of angioregulatory mediators, and inhibitors of both the PI3K and mTOR pathway had anti-proliferative effects on stromal cells and resulted in decreased release of these angioregulatory mediators. PI3K and mTOR inhibitors can decrease constitutive cytokine release both by AML and stromal cells, suggesting potential direct and indirect antileukemic effects.

## INTRODUCTION

Acute myelogenous leukemia (AML) is a heterogeneous malignancy characterized by clonal proliferation and accumulation of immature myeloblasts in the bone marrow [1, 2]. The overall disease-free survival is only 40-50% even for younger patients below 60-65 years of age who receive the most intensive chemotherapy [2], whereas long-term disease-free survival is uncommon for elderly patients and patients unfit for intensive therapy due to comorbidity. New and less toxic therapeutic strategies are therefore strongly needed in AML [3], and one alternative therapeutic strategy is inhibition of intracellular signaling through the phosphatidylinositol 3-kinase (PI3K)/Akt/mammalian target of rapamycin (mTOR) pathway [4-6]. This pathway is important for regulation of cellular growth and metabolism and aberrant PI3K/Akt/mTOR signaling is found in several malignancies [7]. Pharmacological targeting of this pathway is therefore considered as a promising therapeutic approach in several cancer types [8-10], including AML [11-14].

Both normal and leukemic hematopoiesis seem to be supported by neighboring stromal cells in the bone marrow microenvironment, including fibroblast-like cells, osteoblasts and endothelial cells [15-18]. This supportive function is mediated through formation of so-called endosteal and vascular niches that may provide a sanctuary for subpopulations of malign cells to evade or circumvent drug-induced death [19]. Endothelial cells may also contribute to leukemogenesis through AML-induced bone marrow angiogenesis with an increased microvasculatory network in the bone marrow [19, 20]. Bone marrow angiogenesis is regulated by the local cytokine network and a shift in the balance between pro- and antiangiogenic mediators in favor of angiogenesis [21]. Thus, the contribution of angiogenesis to leukemogenesis involves continuous interactions between the leukemic cells and the surrounding microenvironment including the stroma, and angiogenesis may then be supported by the release of angioregulatory cytokines both from the leukemic cells and from the nonleukemic stromal cells [19].

Previous experimental studies have used osteosarcoma cell lines with an osteoblastic phenotype [22, 23], well-characterized fibroblast cell lines [24], and normal endothelial cells [18, 23] as standardized experimental models for the study of AML/stromal cell interactions. In this study, we used the same methodological approach to investigate effects of various PI3K/Akt/mTOR inhibitors and report that the constitutive release of angioregulatory mediators by AML and stromal cells is altered.

## RESULTS

### Primary AML cells differ in their constitutive release of angioregulatory mediators

Angiogenesis is important in leukemogenesis and represents a possible therapeutic target in AML [25]. We quantified the constitutive release of 10 angioregulatory cytokines during *in vitro* culture of primary human AML cells derived from 60 unselected patients. The overall results are summarized in Table 2. The majority of patients showed detectable release of CXCL8, CXCL10, Ang-1, HGF and MMP-9, but the levels showed a wide variation between individual patients. CXCL8 levels (median level 12 002 pg/mL) were generally higher than the other cytokine levels, but HGF was usually also released at relatively high levels (median level 409 pg/mL).

**Figure 1:**
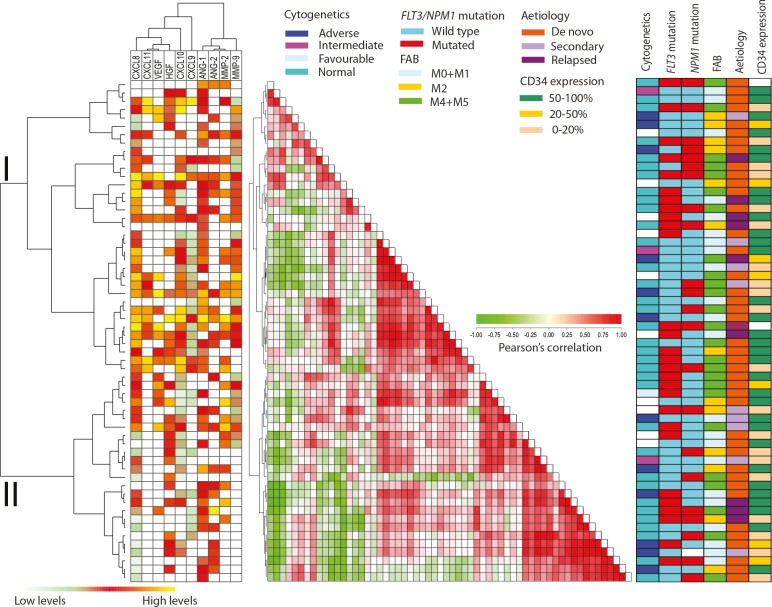
Constitutive release of angioregulatory soluble mediators by primary human AML cells: unsupervised hierarchical cluster analysis (left), distance matrix analysis (middle) and comparison with clinical data (right) The leukemic cells were derived from 60 consecutive patients. For each mediator the concentrations were converted to percent of the maximum value obtained for the whole cohort and this value was then log(2) converted. The Pearson's correlation as distance measure and unweighted pair group method with arithmetic mean linkage was used to create a heatmap with additional unsupervised hierarchical clustering analysis. (LEFT) This panel shows the expression profile where low expression is marked with green and high expression with red to yellow. White represents undetectable values. The hierarchical clustering identified two main patient subsets referred to as cluster I and II (see Table 3). (MIDDLE) The correlation visualization with distance matrix displays the pairwise correlation between the 60 samples; deeper red or green colors indicate a higher positive/negative correlation between the 10 mediators in each sample. (RIGHT) The right panel shows characteristics for each individual patient (genetic abnormalities, morphological differentiation and etiology).

We performed an unsupervised clustering of the 60 patients with regard to their angioregulatory profile, i.e. their constitutive cytokine release (Fig. 1). The values were first normalized by dividing with the maximum release value, and the angioregulatory mediators then formed two major clusters including (i) the proangiogenic HGF, VEGF and CXCL8 together with the antiangiogenic chemokines CXCL9-11 and (ii) Ang-1/2 together with MMP-2/9. The patients could also be divided into two major clusters, the main differences between them being increased release of CXCL8, CXCL9 and Ang-1 in the upper cluster I (Table 3). Thus, the difference in angioregulatory profiles between individual patients is mainly caused by differences in their chemokine release (Table 3), and quantitatively major differences between CXCL8 release was observed between the two clusters. The two clusters did not differ with regard to AML etiology (de novo versus secondary/relapse) differentiation (FAB classification or CD34 stem cell marker expression), cytogenetic abnormalities, and/or frequency of *NPM1* or *FLT3* mutations (Table 3).

### Both PI3K and mTOR inhibitors can alter the constitutive release of angioregulatory mediators by primary human AML cells

We investigated the effects of two mTOR inhibitors (rapamycin 1 μM, temsirolimus 0.1 μM) and two PI3K inhibitors (GDC-0941 1 μM, 3-MA 1 mM) on the release of 10 angioregulatory mediators. Levels of mediators released by AML cells are shown in Table 2 and the results from the statistical analyses are summarized in Table 4. The mTOR inhibitors rapamycin and temsirolimus showed similarities in their inhibitory profile with a strong reduction of CXCL10, HGF and MMP-9 levels corresponding to p-values ≤0.002, whereas there was no significant reduction of the high CXCL8 levels. In contrast, the pan-PI3K inhibitor GDC-0941 caused a statistically significant reduction of all mediators, although the reduction of CXCL9, CXCL11 and VEGF was relatively small. The PI3K-class III inhibitor 3-MA had an intermediate effect and caused a strong inhibition of CXCL9-11, HGF, Ang-1/2 and MMP-2. Thus, even though these pharmacological agents inhibit various steps in the same intracellular signaling pathway, they differ in their effects on the constitutive cytokine release and the PI3K inhibitors then cause a more extensive alteration of the angioregulatory profile than mTOR inhibitors.

**Figure 2:**
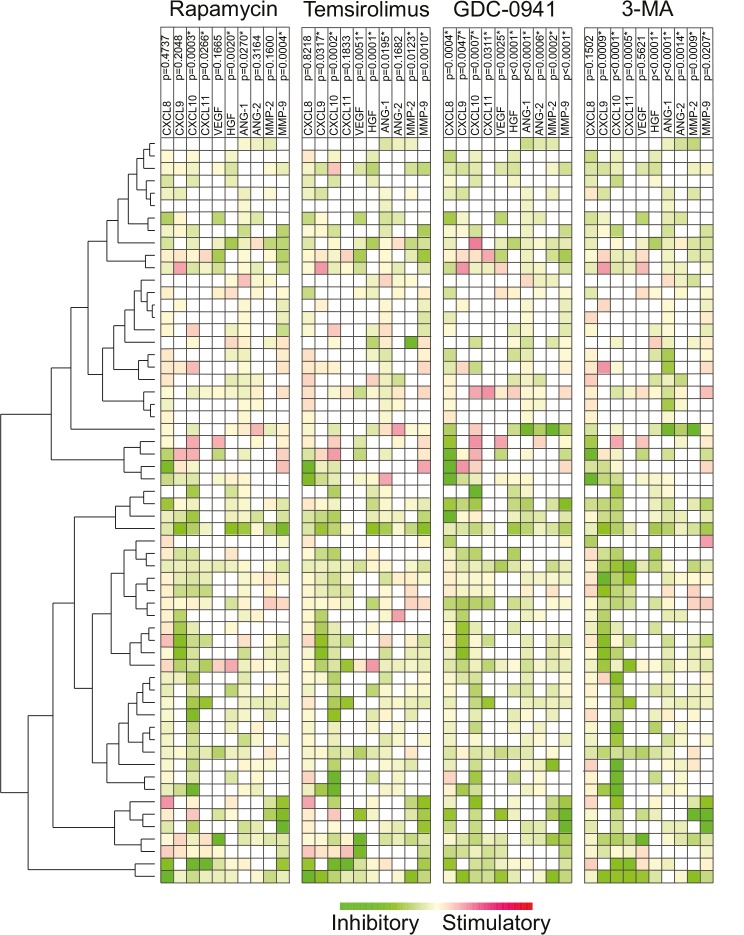
The effect of mTOR (rapamycin, temsirolimus) and PI3K inhibitors (GDC0941, 3-MA) on the constitutive release of angioregulatory mediators by primary human AML cells Leukemic cells from 60 consecutive patients were examined. For each mediator the concentration in treated cultures were divided with the concentration in control cultures before values were log(2) converted. The effects of rapamycin 1 μM, temsirolimus 0.1 μM, GDC-0941 1 μM and 3-MA 1 mM on the release of 10 different angioregulatory mediators were determined. The Euclidian correlation test with complete linkage was used to make a heatmap with additional unsupervised hierarchical clustering analyses. The patients could be divided into two main clusters based on the overall pharmacological effects on the release of the soluble mediators. The green color indicates decreased and red color increased mediator levels. The mediators are listed at the top together with the corresponding p-values when the overall pharmacological effects for the whole patient population were analyzed. * indicates a significant reduction in concentration.

We then performed a clustering analysis of the 60 patients based on the pharmacological effects on the constitutive release of the 10 mediators (Fig. 2). Two major patient clusters were found, and these two clusters differed especially with regard to the pharmacological inhibition of constitutive CXCL9-11 release, whereas the pharmacological effects on release of the other cytokines showed a wide variation and did not differ significantly between the two major clusters.

Finally, we also performed an unsupervised clustering for the 60 AML patients based on the pharmacological effects on release of angioregulatory mediators. The cytokine clustering showed similarities between the four inhibitors and confirmed the observations described above (see Fig. 1): (i) the chemokines clustered separately from the other mediators (ii) VEGF clustered together with HGF, the two angiopoietins clustered together, and the two MMPs clustered together (data not shown).

### Association between protein and mRNA levels for angioregulatory cytokines

**Figure 3:**
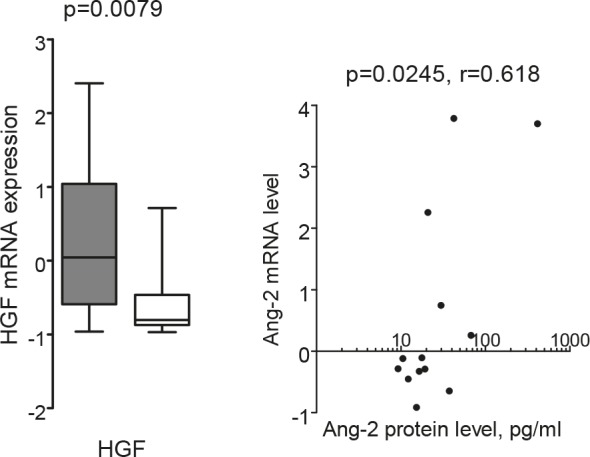
Associations between mRNA levels and supernatant protein levels for HGF and Ang-2 mRNA levels in primary AML cells were compared with proteins levels measured after AML cell culture for 24 hours. (LEFT) Both mRNA and supernatant protein levels were available for 31 patients, and mRNA HGF levels differed significantly when comparing the 19 patients with detectable constitutive HGF secretion (grey box) with the 12 patients showing no detectable HGF release (white box) (Mann-Whitney U-test, p=0.0079). (RIGHT) mRNA Ang-2 levels showed a significant correlation with the Ang-2 protein concentration in culture supernatants of AML cells derived from 13 patients (p=0.0245, r=0.618, Pearson's correlation).

We used microarray analysis to examine mRNA levels in non-cultured leukemia cells for 31 of the 60 AML patients investigated for their constitutive cytokine release. We performed both (i) correlation analysis between detectable cytokine protein levels and mRNA levels and (ii) comparison of mRNA levels for patients with detectable and undetectable cytokine levels. For most of the angioregulatory mediators we did not find any significant correlations between protein/mRNA levels or difference in mRNA levels between patients with detectable and undetectable mediator release. However, two exceptions were seen: (i) patients with undetectable HGF release had a significantly lower HGF-mRNA expression (p=0.0079, Mann-Whitney U-test, Fig. 3); and (ii) Ang-2 mRNA expression showed a significant correlation with supernatant protein levels (p=0.0245, r=0.618, Pearson's correlation, Fig. 3). Thus, only these two cytokines show evidence for a regulation of protein expression at the mRNA level.

### Pharmacological targeting of the PI3K/Akt/mTOR pathway has antiproliferative effects on stromal cells

**Figure 4:**
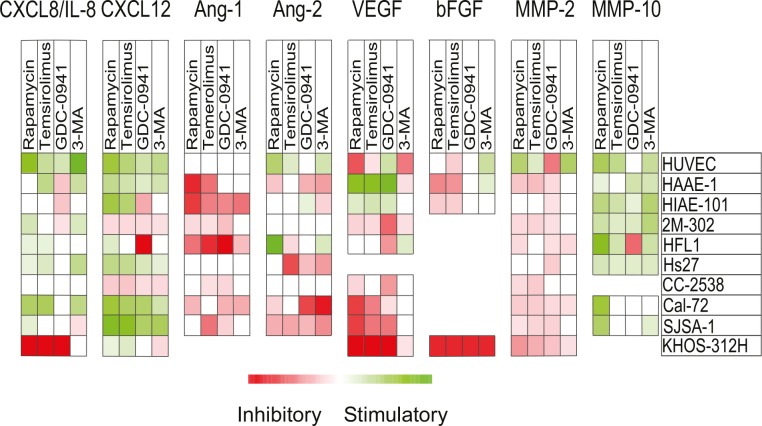
Antiproliferative effects of mTOR and PI3K inhibitors on stromal cells The effects of mTOR (rapamycin, temsirolimus) and PI3K inhibition (GDC-0941, 3-MA) on *in vitro* proliferation of 11 different stromal cell populations was investigated using the ^3^H-thymidine incorporation assay. Detectable proliferation was defined as >1000 cpm. Relative proliferation in drug-treated cultures versus the corresponding drug-free control cultures was converted to log(2) values. The different inhibitors and their concentration (μM) are shown at the top and the cell type is shown in the far right column. The heatmap shows the effects of the different inhibitors on proliferation, i.e. red color indicates decreased growth and green color growth enhancement.

Drug effects on stromal cell proliferation were examined using the ^3^H-thymidine incorporation assay. All stromal cells were treated with drugs over a wide concentration range and dose-dependent antiproliferative effects were observed (Fig. 4). However the effects varied between different cell types and between different drugs:

The mTOR inhibitors rapamycin and temsirolimus generally showed a weaker maximal inhibitory effect than the PI3K inhibitors. These two drugs showed very similar inhibitory effects on stromal cell proliferation with only minimal differences between drugs.

The PI3K inhibitors demonstrated a significant decrease in proliferation only for the highest concentration used in this study. The specific class I PI3K inhibitor GDC-0941 showed a 40 % inhibition for all cell lines at the highest concentration, whereas the class III inhibitor 3-MA caused a similar inhibitory effect but only for certain cell lines.

The different cell lines also varied in their susceptibility to the pharmacological interventions. HUVECs showed less susceptibility to pharmacological interventions, whereas the osteoblastic Cal72 and the fibroblast HFL1 cell line also had a slightly more resistant profile.

Thus, all four drugs show inhibitory effects on stromal cell release of various cytokines at drug concentrations that also have similar inhibitory effects in primary human AML cells.

### Pharmacological interventions have diverse effect on cytokine release from bone marrow stromal cells

We also investigated the effects of pharmacological intervention on the cell release of angioregulatory mediators after treatment with rapamycin 1 μM, temsirolimus 0.1 μM, GDC-0941 1 μM and 3-MA 10 mM. These concentrations were selected because the drugs then showed a clear antiproliferative effect for the majority of susceptible cell lines. For ten investigated stromal cell lines/primary cells, the pharmacological effects on the constitutive release of 13 angioregulatory mediators were examined including CXCL8-12, Ang-1/2, VEGF, HGF, bFGF MMP-2, MMP-9 and MMP-10 (Table 5) and the results were compared with the effects on AML cells (see Table 4):

Disruption of the PI3K/Akt/mTOR pathway generally increased the release of CXCL12 and CXCL8 by stromal cells, except for inhibitory effects observed for GDC-0941. CXCL8 was released at high levels by primary AML cells for most patients and only GDC-0941 inhibited the release.

The antiangiogenic CXCL9, CXCL10 and CXCL11 were not released at detectable levels by any stromal cell types. All three antiangiogenic mediators were released by AML cells and all PI3K/mTOR inhibitors generally inhibited their release.

The release of Ang-1 and to a lesser degree Ang-2 was inhibited by the PI3K/mTOR inhibitors both for stromal and AML cells.

The inhibitors had divergent effects on VEGF release both for stromal and AML cells.

HGF was not released by stromal cells, but was commonly released by AML cells, and treatment with the four PI3K/mTOR inhibitors then reduced this release.

Stromal cells released detectable MMP-2 and MMP-10, but only undetectable or low levels of MMP-9. The PI3K/mTOR inhibitors generally decreased MMP-2 levels, but increased MMP-10 release by stromal cells. In contrast, AML cells released MMP-2 and MMP-9 and levels were decreased by treatment with all PI3K/mTOR inhibitors.

Thus, PI3K/mTOR inhibitors affect stromal cell cytokine release at the same concentrations that affect the release by the primary AML cells, and the drugs seem to have an overall proangiogenic effect on the chemokine release (maintained high CXCL8, decreased CXCL9-11); this effect is further supported by increased MMP-10 release by stromal cells. The increased stromal cell release of CXCL12 may also stimulate leukemogenesis through effects on growth, survival and migration [26]. These AML-stimulating effects are counteracted by decreased HGF, Ang-1, VEGF and MMP-2 levels. The overall effects can only be explained by a specific effect of pharmacological PI3K-Akt-mTOR inhibition on the cytokine release; the effects cannot be explained by a nonspecific effect of decreased proliferation/viability because then a general decrease in the levels of all cytokines would be expected. It is thereby difficult to predict the final effect of PI3K/Akt/mTOR inhibition on bone marrow angioregulation as the effect will probably vary between patients.

## DISCUSSION

The PI3K/Akt/mTOR pathway is implicated in leukemogenesis [27], and has emerged as a potential therapeutic target in AML [6, 7, 14]. Furthermore, several studies suggest a role of angiogenesis in human AML, and this process is regulated by various angioregulatory soluble mediators derived both from the leukemic cells and from the neighboring stromal cells in the bone marrow microenvironment [15, 18, 28]. In the present study we show that inhibitors of the PI3K and mTOR pathway may affect leukemogenesis through inhibition of the constitutive release of angioregulatory mediators both when investigating primary AML cells derived from a large group of unselected patients [29] as well as bone marrow stromal cells [23, 24]. The effects on stromal cells were detected at the same drug concentrations as for the AML cells.

As explained in Material and methods the doses of the various drugs used in our present experiments were selected based on the observations in previous studies and pilot dose-response experiments with primary human AML cells. Our intention was to investigate whether these drugs have simultaneous effects both on leukemic and stromal cells and thus mediate their effects on AML cells both through direct and indirect effects (i.e. via neighboring stromal cells). For this reason we used the same concentrations both in AML and stromal cell experiments. The similarities between the various drugs with regard to their functional effects both in leukemic and stromal cells further support that the effects are mediated through common targets (i.e. the PI3K-Akt-mTOR pathway) and not through off-target effects, and this is also consistent with previous experiments [30-40].

Angiogenesis is defined as the formation of new capillaries from pre-existing blood vessels, and the associations between levels of angiogenic mediators and survival after intensive chemotherapy suggest that angioregulation is also important for chemosensitivity in human AML [41-44]. Several mediators contribute to angioregulation in AML. Firstly, primary AML cells release several pro- (e.g. CXCL8) and antiangiogenic (e.g. CXCL9-11) chemokines, but the chemokine release profile differs between patients [45]. Secondly, AML cells release several MMPs, and MMP-9 seems to be associated with chemokine release, in contrast to MMP-2 which possibly is associated with prognosis after chemotherapy [41]. Finally, other important angioregulatory cytokines that seem to be associated with prognosis in human AML are the Ang-1/2 system, HGF and VEGF [15, 43, 44, 46].

In the present study, we analyzed the constitutive release profile for 10 angioregulatory mediators commonly released by primary human AML cells [47], and we compared the release for 60 unselected patients. Relatively high levels of proangiogenic CXCL8 were detected for almost all patients and this chemokine clustered separately, whereas the antiangiogenic CXCL9-11 clustered together and usually showed lower levels than CXCL8 and for most patients CXCL10 levels were higher than CXCL9/11 levels. These variations in constitutive chemokine release were the major contributors to the classification of patients into two major clusters in the hierarchical cluster analysis based on constitutive release of angioregulatory mediators (Fig. 1). Finally, we observed a wide variation in the pharmacological effects on constitutive mediator release between patients, but the inhibitory effects on chemokine release were most important for the classification/clustering of patients into two major subsets based on effects of PI3K/Akt/mTOR inhibition on the constitutive release of angiogenic mediators (Fig. 2). Finally, clinical or biological characteristics of our patients showed no association with the clustering based on constitutive release of mediators alone or pharmacological effects on the constitutive release (Fig. 1 and Fig. 2).

Bone marrow stromal cells contribute to the local cytokine network and can thereby regulate leukemic hematopoiesis both through direct effects on the malignant cells and through indirect effects via other cells in the bone marrow microenvironment, e.g. the constitutive release of angioregulatory mediators by various stromal cells can affect leukemogenesis through effects on endothelial cells and thereby stimulate angiogenesis and disease development as suggested by previous experimental studies [15, 16, 18, 20, 23, 28]. Potential new therapeutic agents should therefore be evaluated both for their direct effects on AML cells and also for their indirect antileukemic effects through nonleukemic stromal cells. Our present results suggest that such indirect pharmacological effects on stromal cells may contribute to the antileukemic effects of PI3K/Akt/mTOR inhibitors.

The PI3K and mTOR inhibitors evaluated in this study inhibited growth of bone marrow stromal cells, suggesting that they have indirect antileukemic effects mediated via the stromal cells. Previous studies have shown that these drugs have antiangiogenic and thereby anticancer effects in human malignancies [48, 49], including by decreased expression of both VEGF and its receptors [48, 50]. However, our results show that disruption of the PI3K/Akt/mTOR pathway can affect angiogenesis not only via the VEGF system but also through altered release of several angioregulatory mediators released both by AML as well as various stromal cells. Such indirect effects may thus contribute to the antileukemic activity of these drugs [51]. Finally, another effect of these drugs seems to be altered leukemia cell migration [52] that may be caused by effects on stromal cells cytokine release and possibly affect localization of AML cells to the growth-enhancing stem cell niches.

**Figure 5:**
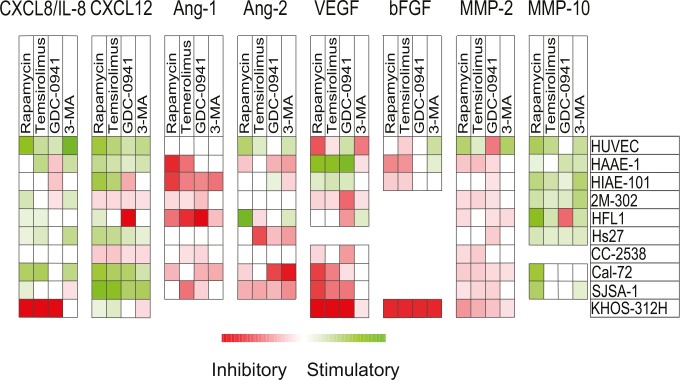
Effects of mTOR and PI3K inhibitors on constitutive release of angiogenic mediators by stromal cells The effects of mTOR (rapamycin, temsirolimus) and PI3K inhibition (GDC-0941, 3-MA) on *in vitro* constitutive mediator release was investigated for 10 different stromal cell populations. Cell supernatants were harvested from stromal cell cultures before confluence was reached. Levels of each mediator were determined using ELISA, and relative values (levels in drug-treated cultures divided by levels in corresponding control cultures) were log(2) converted. Squares are omitted where no detectable levels of mediators were measured in control/treated cultures. The various stromal cell populations examined are indicated in the right column.

The final effect of these drugs on angioregulation depends on their effects on the local balance between pro- and antiangiogenic mediators. Our overall results suggest that these drugs often have a relatively weak effect on proangiogenic CXCL8 that is released at high levels by AML cells for most patients; a strong inhibitory effect on this proangiogenic mediator was only seen for a subset of patients and an increased release was often seen for the stromal cells. The drugs had divergent effects on several other mediators (see Table 5 and Fig. 5) and it is possible that other growth factors/cytokines involved in AML-stroma interactions may be targeted by PI3K/mTOR inhibitors. It is therefore difficult to predict the final overall effect of these drugs on bone marrow angioregulation, though in our opinion it seems most likely that the final effect will differ between individual patients, and for many patients antiangiogenic effects do not seem to be a major contributor to the antileukemic effects seen by the investigated PI3K/Akt/mTOR inhibitors and may also involve microenvironment-mediated drug-resistance [53, 54].

To conclude, our present results suggest that disrupting PI3K/Akt/mTOR signaling can alter the local bone marrow cytokine network both through effects on leukemia cells as well as various stromal cells, and these alterations may directly affect the leukemic cells or indirectly affect leukemogenesis through altered regulation of bone marrow angiogenesis.

## MATERIAL AND METHODS

### AML patients and cell preparation

The study was approved by the local Ethics Committee (Regional Ethics Committee III, University of Bergen, Norway) and samples collected after written informed consent. The study included a total of 60 consecutive adult AML patients with high peripheral blood blast counts (>7 × 10^9^/L). This selection of patients and the analysis of *FLT3* and *NPM1* mutations have been described previously [29, 46]. The major characteristics of the 60 patients (median age 65.5 years, range 24-84 years) are summarized in Table 1. AML cells were isolated from peripheral blood by density gradient separation (Lymphoprep, Axis-Shield, Oslo, Norway) and the final leukemic cell populations studied contained at least 95% leukemia blasts. The cells were stored in liquid nitrogen [29].

**Table 1:** Main characteristics of the 60 AML patients included in the study

Patient characteristics		Number (percent)
Demographic data and disease history		
Gender (numbers)	Male/female	33 (55)/27(45)
AML history	De novo	41 (68.3)
	Secondary	9 (15.0)
	Relapsed	10 (16.7)
AML cell differentiation		
FAB classification	M0-1	22 (36.6)
	M2	13 (21.7)
	M4-5	25 (41.7)
CD34 expression¶	0-20%	20 (33.3)
	20-50%	7 (11.7
	50-100%	31 (51.7)
Genetic abnormalities*		
Cytogenetics	Favorable	1 (1.7)
	Intermediate	3 (5.0)
	Adverse	12 (20)
	Normal	34 (56.7)
FLT3†	ITD	25 (41.7)
	TKD	4 (6.7)
NPM1	Mutated	22 (36.7)

¶ )Presented as the percentage of positive cells in flow cytometric analysis.

* )Favorable cytogenetics includes one patient with inv(16), intermediate includes three patients with either + 8, +14 or + 13, adverse includes eight patients with multiple (≥ 3) abnormalities, two patients with -7 and two patients with -5.

† ) One patient had both a FLT3-ITD and a FLT3-TKD mutation.

**Table 2:** Constitutive release of angioregulatory factors by primary human AML cells derived from 60 consecutive patients The levels are presented as median and range.

Angiogenic factors	Number of patients with detectable release	Cytokine levels (pg/ml) for patients with detectable release
CXCL8	54	12 002 (14.2-189 169)
CXCL9	30	27.5 (6.9-18 368)
CXCL10	46	191 (2.78-25 468)
CXCL11	16	246.8 (40.2-2645)
Ang-1	45	121 (18.6-4337)
Ang-2	25	19.0 (9.3-415)
VEGF	18	34.4 (5.4-197)
HGF	31	409 (40.0-7736)
MMP-2	26	1530 (230-10 900)
MMP-9	38	4290 (250-379 400)

**Table 3:** Biological characteristics of the two major patient subsets identified by cluster analysis of the constitutive release of soluble mediators (cytokines and MMPs, see right part of Fig. 1)

Mediator	Cluster I	Cluster II	p-value
CXCL8	23 718 (3.5-289 169)	413 (3.5-43 561)	0.0002
CXCL9	12.7 (3.8-18 368)	3.8 (3.8-49.8)	0.0066
CXCL10	540 (1.7-25 468)	10.0 (1.7-574)	0.0004
CXCL11	13.9 (13.9-2645)	13.9 (13.90-13.9)	0.0060
Ang-1	111 (3.5-4337)	73.3 (3.5-1170)	n.s
Ang-2	8.3 (8.3-67.4)	8.3 (8.3-415)	n.s
VEGF	5.0 (5.0-197)	5.0 (5.0-75.4)	n.s
HGF	40.0 (40.0-7736)	112 (10.0-2440)	n.s
MMP-2	160 (160-10 900)	160 (160-5660)	n.s
MMP-9	4090 (160-379 400)	160 (160-21 860)	<0.0001

The levels of the soluble mediators are presented as median concentrations (pg/ml), the variation range is given in parenthesis. The Mann-Whitney U test was used for the statistical comparison (ns; not significant).

**Table 4:** Effects of mTOR and PI3K inhibitors on the constitutive release of angioregulatory mediators by primary human AML cells

Soluble mediator	Rapamycin	Temsirolimus	GDC-0941	3-MA
CXCL8	96.5 % 0.4737	103.0 % 0.8218	66.0 % 0.0004	93.5 % 0.1502
CXCL9	76.0 % 0.2048	69.0 % 0.0317	50.0 % 0.0047	32.5 % 0.0009
CXCL10	50.5 0.0003	52.0 0.0002	53.0 0.0007	29.0 0.0001
CXCL11	70.5 0.0226	81.0 0.183	56.5 0.0311	35.0 0.0005
Ang-1	89.0 0.0270	89.0 0.0195	76.0 <0.0001	71.0 <0.0001
Ang-2	97.0 0.3164	88.0 0.1682	85.0 0.0006	79.0 0.0014
VEGF	64.0 0.1665	41.0 0.0051	51.0 0.0255	86.0 0.5621
HGF	75.0 0.0020	72.0 0.0001	57.5 <0.0001	57.0 <0.0001
MMP-2	96.0 0.1640	70.5 0.0123	54.3 0.0002	58.5 0.0009
MMP-9	64.5 0.0004	60.0 0.0010	59.0 <0.0001	83.5 0.0207

The results are presented as the median % inhibition (upper value) of mediators released after drug treatment compared to levels in control cultures without drugs. The corresponding p-value (lower value) is shown where statistically significant differences are marked in bold. The drugs were tested at the following concentrations: rapamycin 1.0 μM, temsirolimus 0.1 μM, GDC-0941 1.0 μM and 3-MA 1.0 mM).

**Table 5:** Constitutive release of angioregulatory soluble mediators by different stromal cell lines

CELL TYPE	CXCL8/IL-8 pg/ml	CXCL12 pg/ml	Ang-1 pg/ml	Ang-2 pg/ml	VEGF pg/ml	bFGF pg/ml	MMP-2 ng/ml	MMP-10 pg/ml
Endothelial cells (HUVEC)	12.0	42.3	171.2	300.0	325.3	4.7	7.2	1504.4
Endothelial cells (HAAE-1)	573.1	68.3	193.9	85.3	9.7	23.4	30.3	668.1
Endothelial cells (HIAE-101)	540.2	48.4	127.1	76.2	129.1	71.4	16.2	221.4
Bone marrow stromal cells (2M-302)	797.8	559.1	3423.1	598.4	730.3	nd	22.7	16.0
Fibroblast (HFL1)	79.6	109.7	229.0	48.0	11.9	nd	6.9	16.0
Fibroblast (Hs27)	320.9	49.6	72.8	160.3	nd	nd	11.4	285.1
Osteoblasts (CC-2538)	676.5	775.5	2473.6	676.4	563.8	nd	25.7	n.d
Osteoblastic cells (Cal-72)	12.1	37.4	672.9	19.9	671.1	nd	36.2	9.9
Osteoblastic cells (SJSA-1)	1361.8	24.8	46.5	184.5	153.5	nd	18.2	64.2
Adherent osteosarcoma cell line (KHOS-312H)	41.2	104.2	nd	nd	781.7	15.8	8.5	nd

The table presents the levels in culture supernatants. nd; not detectable. None of the stromal cells released detectable levels of CXCL9-11, MMP-9 or HGF.

### Normal stromal cells and cell lines

All human cell lines and primary cells were grown and expanded in their recommended media (distributor's information) and obtained from either Deutsche Sammlung von Zellkulturen und Mikroorganismen (DSZM, Braunschweig, Germany), American Type Culture Collection (ATCC Vanassas, MA) or Lonza (Cambrex BioScience, Walkersville, MD) as stated below. Primary cells were utilized at passages 2 to 4. The following cell types were included in the study:

Human Umbilical Vein Endothelial Cells (HUVEC) (ATCC no C2517A) and the two arterial endothelial cell types; HAAE-1 (ATCC no CRL-2472) and HIAE-101 (ATCC no CRL-2478).

Normal bone marrow stromal cells (2M-302; Lonza) and the fibroblast cell lines HFL1 (ATCCs no CCL-153) and Hs27 (ATCC no CRL-1634).

Normal osteoblasts (Clonetics, Normal Human Osteoblast Cell System NHOst, CC-2538) and the two osteoblastic osteosarcoma cell lines Cal72 (DSZM) and SJSA-1 (ATCC no CRL-2098).

We also included SAOS (ATCC no HTB-85), SKES-1 (ATCC no HTB-86), U2OS (ATCC no HTB-96), which are osteoblastic sarcoma cell lines with an epithelial phenotype, and the adherent Ewing sarcoma cell line KHOS-312H (ATCC no CRL-1546).

### Pharmacological agents

All the drugs were tested in initial dose-response experiments for primary human AML cells derived from 15 unselected patients; we then used a highly standardized ^3^H-thymidine incorporation assay for cytokine-dependent proliferation as published in previous methodological studies [45, 55-57]. All drugs inhibited AML cell proliferation at the concentrations used in the present experiments. Stock solutions of rapamycin and temsirolimus were prepared in dimethylsulphoxide (DMSO), and pilot experiments showed that DMSO at the concentrations reached in the experiments did not affect AML cell proliferation. Thus, all drugs were tested at concentrations showing biological effects on primary AML cells, and these effects were not caused by other constituents of the drug preparation. We wanted to investigate whether the drugs have combined direct and indirect (i.e. effects mediated via stromal cells) antileukemic effects, and for this reason we used the same drug concentrations in stromal cell experiments as in the AML cell studies.

#### Rapamycin

Rapamycin (LC Laboratories, Woburn, MA) inhibits both mTORC1 and mTORC2 [30]. Initially we investigated the effect of rapamycin as a tenfold dilution between 0.01 and 10^5^ nM. All concentrations caused a similar and statistically significant inhibition of AML cell proliferation corresponding to 68 % (0.01 and 10^4^ nM) to 77 % (10^3^ nM) of the drug-free controls. Studies in myeloma cells have also shown a similar antiproliferative effect when rapamycin was tested over a similar concentration range [31], and previous *in vitro* studies of primary human AML cells suggest that some patients show no inhibition of mTOR activity when testing rapamycin at ≤20 nM but with a maximal effect being reached at rapamycin >50 nM [32]. Based on these overall data we used rapamycin 100 nM in our experiments.

#### Temsirolimus

This rapamycin-derivative (LC Laboratories, Woburn, MA, US) causes an inhibition of both mTORC1 and mTORC2 when tested at high concentrations corresponding to 1250 nM [33], whereas detailed studies in cancer cell lines have demonstrated that for concentrations up to 500 nM temsirolimus is an mTORC1-specific inhibitor [34]. We used temsirolimus at 100 nM; this concentration is within the range corresponding to selective mTORC1 inhibition, it has an antiproliferative effect in primary human AML cells and it has been used in previous experimental studies [4, 34].

#### GDC-0941

This PI3K class I specific inhibitor (Axon Medchem BV, Groningen, Netherlands) was tested at 100 nM [35, 36].

#### 3-methyladenine (3-MA)

This is an inhibitor of both class I and class III PI3Ks and its biological effects are caused by effects on mTOR as well as other downstream targets of Akt [37-40] 3-MA (Sigma-Aldrich Corp. St. Louis, MO) was tested at 1 mM [37-40] and 10mM.

### Analysis of constitutive release of soluble mediators by primary AML cells and stromal cells

#### Culture of AML cells

The methodological approach for analysis of constitutive cytokine release has been characterized in detail in previous methodological studies [45, 55-57]. Briefly, AML blasts (2 × 10^6^ cells in 2 ml) were cultured in 24-well culture plates (Costar 3524; Cambridge, MA) for 48 hours in StemSpan SFEM serum-free medium (referred to as StemSpan; Stem Cell Technologies; Vancouver, BC, Canada) supplemented with 100 μg/ml of gentamicin. Supernatants were collected after 48 hours of culture at 37°C in a humidified atmosphere of 5% CO_2_.

#### Stromal cell culture

The culture conditions for the stromal cells have been characterized in detail previously [23]. Briefly, stromal cell populations were cultured in Stem Span supplemented with 10% heat-inactivated fetal calf serum (FCS) (BioWhittaker, Veviers, Belgium) and 100 μg/ml of gentamicin in 24-well culture plates (Costar 3524), 2 × 10^4^ cells were then seeded per well. Supernatants were harvested before the cells reached confluence.

### Analysis of soluble mediator concentrations in supernatants

Mediator levels were determined using Quantikine enzyme-linked immunosorbent assays (ELISA) (R&D Systems, Minneapolis, MN). All assays were performed strictly according to the manufacturer's instructions. The minimal detectable levels were CXCL8 3.5 pg/mL, CXCL9 3.8 pg/mL , CXCL10 1.7 pg/mL, CXCL11 13.9 pg/mL, CXCL12 18 pg/mL, basic fibroblast growth factor (b-FGF) 3.0 pg/mL, vascular endothelial growth factor (VEGF) 5.0 pg/mL, hepatocyte growth factor (HGF) 40 pg/mL, angiopoietin (Ang)-1 3.5 pg/mL, Ang-2 8.3 pg/ml, matrix metalloprotease (MMP)-2 0.16 ng/mL and MMP-9 0.16 ng/mL and MMP-10 4.1 pg/mL.

### RNA preparation, labeling and microarray hybridization

Primary human AML cells were investigated without any further *ex vivo* manipulation after isolation by gradient separation. All microarray experiments were performed using the Illumina iScan Reader, which is based upon fluorescence detection of biotin-labeled cRNA. Frozen primary AML cells were thawed and total RNA was prepared as described previously [58]. Total RNA 300 ng from each sample was reversely transcribed, amplified and biotin-16-UTP-labeled using the Illumina TotalPrep RNA Amplification Kit (Applied Biosystems/Ambion, Foster City, CA). Amount and quality of the biotin-labeled cRNA was controlled both by NanoDrop spectrophotometer analysis and by using the Agilent 2100 Bioanalyzer before 750 ng cRNA was hybridized to the HumanHT-12 v4 Expression BeadChip. The HumanHT-12 v4 BeadChip targets 47 231 probes derived primarily from genes in the NCBI RefSeq database (Release 38).

### Analysis of stromal cell proliferation

Stromal cells (5 × 10^3^ cells/well) were cultured in flat-bottomed microtitre 96-well plates (NucleonTM Surface, Nunc A/S, Roskilde, Denmark), with 150 μl medium per well. All experiments were prepared in triplicates. Before cells reached confluency, ^3^H-thymidine 37 kBq per well (TRA 310, Amersham, UK) was added in 20 μl of 0.9% NaCl solution and nuclear radioactivity was assayed 24 hours later. Cultures were then transferred to UniFilter-96 well GF/C plates (PerkinElmer Inc., Wellesley, MA), Microscint− scintillation fluid was added (PerkinerElmer Inc.) to air dried plates and radioactivity was measured using a scintillation plate reader (TopCount NXT−, Packard Instruments, PerkinElmer Inc).

### Statistical and bioinformatical analyses

All statistical analyses were performed using the Statistical Package for the Social Sciences (SPSS) v16.0 (SPSS Inc., Chicago, IL) and GraphPad Prism v5 (GraphPad Software, Inc., San Diego, CA), and p-values <0.05 were regarded as statistically significant. The data obtained from the scanning of arrays with the Illumina iScan Reader were analyzed using GenomeStudio and J-Express 2012 software for quality control measures [59]. All arrays within each experiment were quantile normalized to be comparable and then compiled into an expression profile data matrix,. Bioinformatical analyses were performed using the J-Express 2012 analysis suite (MolMine AS, Bergen, Norway) [59]. The normalized values were transformed to logarithmic values (base 2) before analyses, and unsupervised hierarchical clustering was performed with correlation and distance measure.
