# Correction: Pharmacologic targeting of the PI3K/mTOR pathway controls release of angioregulators from primary human acute myeloid leukemia cells and their neighboring stromal cells

**DOI:** 10.18632/oncotarget.14676

**Published:** 2017-01-16

**Authors:** Håkon Reikvam, Ina Nepstad, Øystein Bruserud, Kimberley Joanne Hatfield

**PRESENT: Due to an error during production, Figures 4 and 5 were displayed as duplicates**.

**Correct**: Figures 4 and 5 are correctly displayed below. The authors sincerely apologize for this oversight.

Original article: Oncotarget. 2013; 4(6):830-43. doi: 10.18632/oncotarget.971.

**Figure 4:**
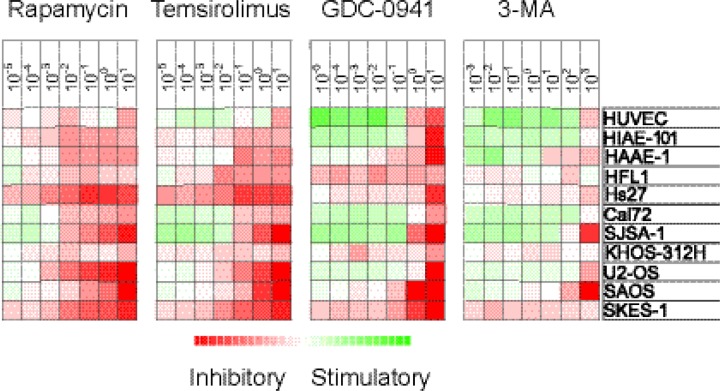
Antiproliferative effects of mTOR and PI3K inhibitors on stromal cells The effects of mTOR (rapamycin, temsirolimus) and PI3K inhibition (GDC-0941, 3-MA) on *in vitro* proliferation of 11 different stromal cell populations was investigated using the ^3^H-thymidine incorporation assay. Detectable proliferation was defined as >1000 cpm. Relative proliferation in drug-treated cultures versus the corresponding drug-free control cultures was converted to log(2) values. The different inhibitors and their concentration (μM) are shown at the top and the cell type is shown in the far right column. The heatmap shows the effects of the different inhibitors on proliferation, i.e. red color indicates decreased growth and green color growth enhancement.

**Figure 5:**
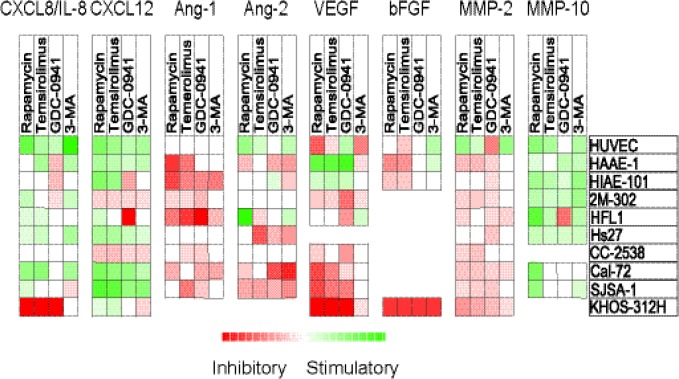
Effects of mTOR and PI3K inhibitors on constitutive release of angiogenic mediators by stromal cells The effects of mTOR (rapamycin, temsirolimus) and PI3K inhibition (GDC-0941, 3-MA) on *in vitro* constitutive mediator release was investigated for 10 different stromal cell populations. Cell supernatants were harvested from stromal cell cultures before confluence was reached. Levels of each mediator were determined using ELISA, and relative values (levels in drug-treated cultures divided by levels in corresponding control cultures) were log(2) converted. Squares are omitted where no detectable levels of mediators were measured in control/treated cultures. The various stromal cell populations examined are indicated in the right column.

